# Coronavirus sampling and surveillance in bats from 1996–2019: a systematic review and meta-analysis

**DOI:** 10.1038/s41564-023-01375-1

**Published:** 2023-05-25

**Authors:** Lily E. Cohen, Anna C. Fagre, Binqi Chen, Colin J. Carlson, Daniel J. Becker

**Affiliations:** 1 Icahn School of Medicine at Mount Sinai, New York, NY USA; 2 Department of Microbiology, Immunology, and Pathology, College of Veterinary Medicine and Biomedical Sciences, Colorado State University, Fort Collins, CO USA; 3 Center for Global Health Science and Security, Georgetown University Medical Center, Washington, DC USA; 4 Department of Biology, University of Oklahoma, Norman, OK USA

**Keywords:** Viral reservoirs, Ecological epidemiology, Data mining

## Abstract

The emergence of SARS-CoV-2 highlights a need for evidence-based strategies to monitor bat viruses. We performed a systematic review of coronavirus sampling (testing for RNA positivity) in bats globally. We identified 110 studies published between 2005 and 2020 that collectively reported positivity from 89,752 bat samples. We compiled 2,274 records of infection prevalence at the finest methodological, spatiotemporal and phylogenetic level of detail possible from public records into an open, static database named datacov, together with metadata on sampling and diagnostic methods. We found substantial heterogeneity in viral prevalence across studies, reflecting spatiotemporal variation in viral dynamics and methodological differences. Meta-analysis identified sample type and sampling design as the best predictors of prevalence, with virus detection maximized in rectal and faecal samples and by repeat sampling of the same site. Fewer than one in five studies collected and reported longitudinal data, and euthanasia did not improve virus detection. We show that bat sampling before the SARS-CoV-2 pandemic was concentrated in China, with research gaps in South Asia, the Americas and sub-Saharan Africa, and in subfamilies of phyllostomid bats. We propose that surveillance strategies should address these gaps to improve global health security and enable the origins of zoonotic coronaviruses to be identified.

## Abstract (web summary)

Systematic review and analysis of pre-pandemic coronavirus surveillance in bats identifies requirements for improvements to surveillance in the future.

## Main

Since the emergence of severe acute respiratory syndrome-associated coronavirus (SARS-CoV) in 2002, coronaviruses (Coronaviridae: Orthocoronavirinae) have been recognized as potential pandemic threats. The group comprises four genera containing an estimated hundreds, or thousands, of viruses^1^. The delta- and gammacoronaviruses are primarily bird pathogens, although they also infect some mammals; notably, porcine deltacoronavirus was reported to infect humans in 2021 (ref. ^2^). The alpha- and betacoronaviruses contain all other known human-infective coronaviruses. Betacoronaviruses include SARS-CoV, Middle East respiratory syndrome-related coronavirus (MERS-CoV) and severe acute respiratory syndrome coronavirus 2 (SARS-CoV-2), all of which have caused morbidity and mortality in humans^3^. While alpha- and betacoronaviruses can infect many different hosts, substantial diversity of coronaviruses occurs in bats, which are probably the ancestral hosts of these coronavirus genera^4,5^. Owing to this, coronaviruses, along with other clades of zoonotic viruses including filoviruses, lyssaviruses and henipaviruses, continue to be extensively monitored in wild bats^6^.

Research into the natural origins of SARS-CoV-2 and continuing interest in coronavirus ecology and evolution have highlighted the value of wild bat surveillance. However, field sampling is often carried out opportunistically in response to concerns about spillover, and capacity for systematic sampling is financially or logistically constrained^7^. For example, comparative analyses of bat filovirus and henipavirus positivity have shown that only a small fraction of studies report longitudinal data, limiting inference into temporal dynamics of infection in bats^6^. Single sampling events can bias prevalence estimates in biologically meaningful ways, for example if sampling is more convenient in one season over another, and may lead to non-randomly missing data. Unlike single sampling studies, spatiotemporal designs can identify seasonal and environmental drivers of viral prevalence and shedding intensity, but they are logistically challenging and often have either spatial or temporal replication but not both^6^.

If the ultimate goal is to explain and predict pathogen spillover—a dynamic process that is driven by geographical and temporal variation in infection prevalence and shedding from reservoir hosts^6,8^, there is a critical need to resolve the relative importance of spatiotemporal, taxonomic and methodological factors (for example, tissues sampled, use of euthanasia, diagnostic method) that may impact virus positivity. Unfortunately, a lack of standardized and aggregated data from disparate studies limits our ability to quantify whether and how these many different factors shape global assessments of coronavirus infection in bats and downstream spillover risk.

To provide baseline data to inform future surveillance efforts, we compiled a standardized global database of infection prevalence estimates using published pre-pandemic coronavirus testing data from wild bat samples and included metadata on bat and viral taxonomy, study methodology, bat demography, bat seasonality and ecological context. We used our database to test several standing hypotheses, including that (1) longitudinal sampling results in higher virus detection rates^6,9^, (2) seasonality affects virus shedding and detection rates^1,10^ and (3) viral detection varies in different sample types^11^. More broadly, we evaluated the global state of coronavirus surveillance in bat hosts before SARS-CoV-2-motivated research efforts.

## Results

### Dataset description

**Fig. 1:**
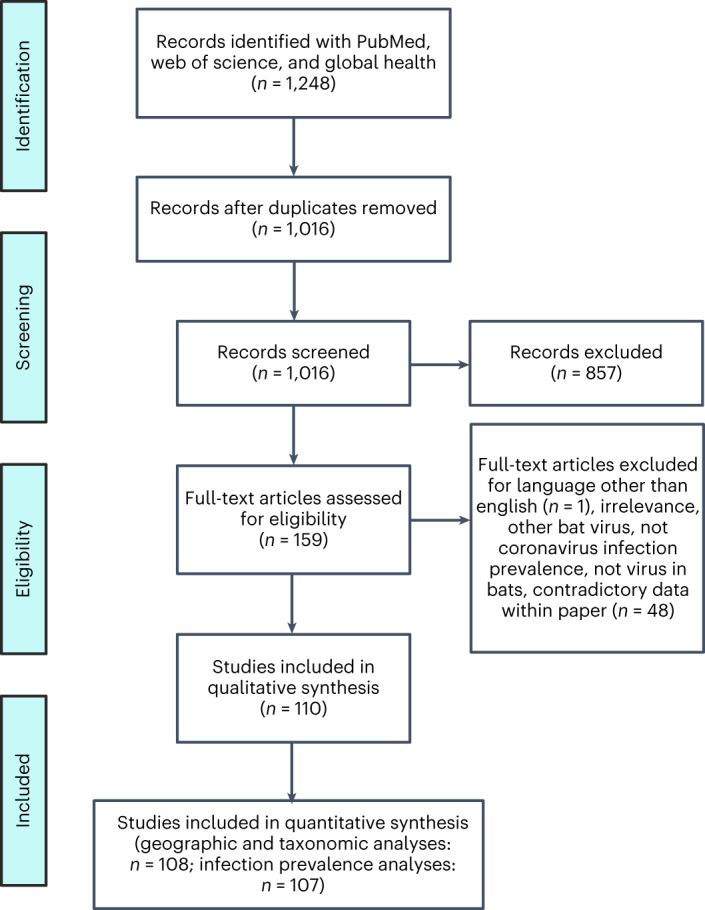
PRISMA reporting for systematic review and meta-analysis.

We first identified global biases in the distribution and intensity of pre-pandemic bat coronavirus surveillance. From publicly available literature published between 2005 and 2020, we recovered 89,752 tests for coronaviruses in bats from 110 studies^12–121^ (Fig. 1 and Supplementary Table 1). Within the pooled-coronavirus genera (alpha- and betacoronavirus) infection prevalence dataset, which comprised data from 107 studies, approximately 95% of studies used PCR targeting the RNA-dependent RNA polymerase (RdRp) gene to detect viruses; other gene targets included subunits of the coronavirus spike protein, the nucleocapsid gene or the envelope protein. Of the 106/107 studies detecting coronaviruses by PCR, approximately 56% used single-round PCR, as opposed to nested PCR or multiple PCR assays in parallel to target different genes in the same RNA sample. More than half of these studies (53.8%) designed their primers using protocols from four studies^11–124^. Of the pooled-coronavirus genera infection prevalence records, 35% was derived from studies that had euthanized bats. Supplementary Table 2 lists the sample types analysed and the associated percentages of positive and zero-infection prevalence. Faecal samples and rectal swabs were the most common samples used to detect coronavirus RNA. Sex and/or reproductive status of bats was only described in 13 of 110 studies in our full database, limiting downstream analyses of sex biases in coronaviruses infection or possible impacts of reproductive stress on viral susceptibility and shedding^8^.

### Spatial bias in coronavirus surveillance

**Fig. 2:**
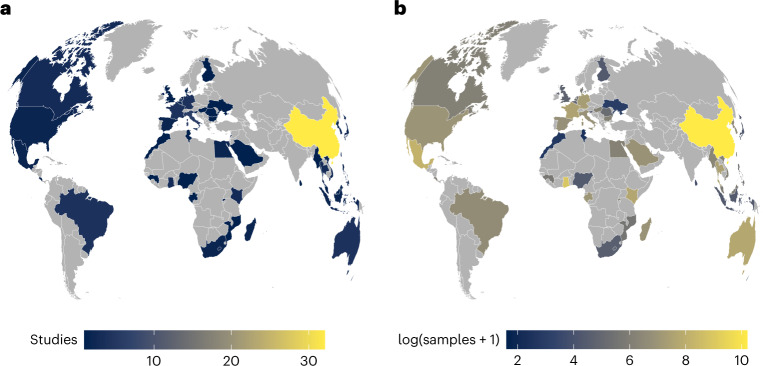
Geographic distribution of bat coronavirus sampling effort. Geographic distribution is defined by the number of studies per country (**a**) and the number of samples tested per country (**b**). Sampled countries varied in having 1 to 32 bat coronavirus studies (**a**), with the number of total samples tested ranging from 4 to 26,051 (**b**). A disproportionate number of bat coronavirus studies and testable samples were conducted and assayed in China, probably reflecting interest in the subgenus *Sarbecovirus* and the risk of future SARS-like virus emergence. Many areas were severely understudied, particularly relative to ecological and evolutionary risk factors for emergence^131^. In particular, sampling in Central and South America, sub-Saharan Africa and Central and South Asia was notably limited.

Before the COVID-19 pandemic, we identified studies reporting sampling of wild bats for coronavirus infection in 52 countries on 6 continents. However, the distribution and frequency of viral surveillance was uneven (Fig. 2). Individual countries had 1 to 32 bat coronavirus studies (Fig. 2a), with the number of total samples tested ranging from 4 to 26,051 (Fig. 2b). Whereas sampling occurred in all North American countries, Central and South America had sparse surveillance. Sampling in sub-Saharan Africa and in Central and South Asia has been inconsistent, with most surveillance carried out in China and in some other regions of Southeast Asia. A generalized linear model (GLM) of binary sampling effort (*χ*^2^ = 13.02, *P* = 0.01, *R*^2^ = 0.04) confirmed that countries in Asia and Europe were marginally more likely to have data on bat coronaviruses than those in the Americas and in Oceania (Supplementary Table 3). We found substantial geographic biases for the relative intensity of sampling, specifically the number of studies (*χ*^2^ = 17.92, *P* = 0.001, *R*^2^ = 0.06) and the number of tested samples (*χ*^2^ = 20671, *P* < 0.001, *R*^2^ = 0.12). Post-hoc comparisons using GLMs revealed that there were more bat coronavirus studies per country in Asia than in Africa or Europe (Supplementary Table 4). Similarly, the greatest contrast in total number of tested bat samples was between Asia and Europe (risk ratio = 4.64), and between the Americas and Europe (risk ratio = 2.11; Supplementary Table 5).

### Taxonomic biases in surveillance

**Fig. 3:**
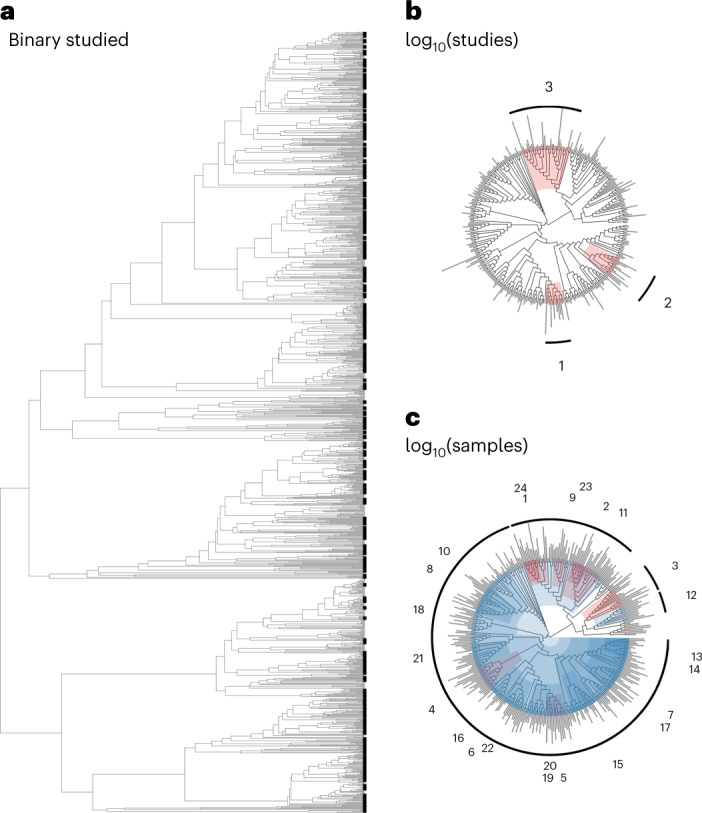
Evolutionary distribution of bat coronavirus sampling effort. Sampling effort is defined as whether a bat species has been sampled (**a**), the number of studies (**b**) and the number of samples tested (**c**). Clades identified by phylogenetic factorization with greater or lesser sampling effort compared with a paraphyletic remainder are shown in red and blue, respectively, alongside clade numbers per analysis. Phylogenetic factorization did not identify any taxonomic patterns in binary sampling effort across the bat phylogeny (**a**), but did identify a number of bat clades within sampled bat species that have been particularly well-sampled for coronaviruses, both in terms of number of studies (**b**; Supplementary Table 8) and number of samples (**c**; Supplementary Table 9, only the first 24 phylogenetic factors are displayed). For analyses of total studies and tested samples, segment length corresponds to the relative degree of sampling effort.

More than 1 in 4 bat species (343 species of the 1,287 included in the most recent bat phylogeny^125^) were sampled in pre-COVID-19 pandemic coronavirus surveillance. Bats have been sampled evenly across the phylogeny (Fig. 3a). Of the 19 bat families included in this phylogeny, 15 had at least 1 member species sampled in our dataset. Unsampled bat families included the Furipteridae, Natalidae, Myzopodidae and Thyropteridae. Indeed, we only identified intermediate phylogenetic signal in binary sampling effort (*D* = 0.86) that departed from both phylogenetic randomness (*P* < 0.001) and Brownian motion models of evolution (*P* < 0.001). Similarly, phylogenetic factorization^126^, a graph-partitioning algorithm based on the bat phylogeny, did not identify any bat clades that differed considerably in their fraction of sampled species. In contrast, we observed stronger taxonomic biases in sampling intensity. The number of studies per sampled species ranged from 1 to 23 (*Miniopterus schreibersii* and *Rhinolophus ferrumequinum*), whereas the number of total samples tested ranged from 1 to 16,499 (*Rhinolophus sinicus*). The number of studies per sampled species showed low phylogenetic signal (*λ* = 0.02) that departed from Brownian motion models of evolution (*P* < 0.001) but not phylogenetic randomness (*P* = 0.56). Phylogenetic factorization did, however, more flexibly identify 3 bat clades with greater mean numbers of studies than the paraphyletic remainder (Fig. 3b): a subclade of the genus *Myotis* (including both European and Asian species), a subclade of the tribe Pipistrellini (including the genera *Pipistrellus* and *Nyctalus*) and a subclade of the family Rhinolophidae (Supplementary Table 8); notably, all highly sampled clades consisted exclusively of Old World bat species.

For the total number of tested samples per species, we instead observed more intermediate phylogenetic signal (*λ* = 0.27) that departed from both Brownian motion models of evolution (*P* < 0.001) and phylogenetic randomness (*P* < 0.001). Accordingly, phylogenetic factorization identified a total of 39 clades with differential intensities of sampling effort, 15 of which had relatively more tested samples and 24 had relatively fewer tested samples (Fig. 3c). The top clades with comparatively fewer total samples included a large portion of the suborder Yangochiroptera; the above-mentioned subclade of the tribe Pipistrellini; members of the phyllostomid subfamilies Stenodermatinae, Glossophaginae and Phyllostominae; and the sister families Rhinolophidae and Hipposideridae; these results suggest a greater number of publications on some of these bat taxa but fewer tested samples. However, smaller subclades of the Hipposideridae and Rhinolophidae families were some of the most heavily sampled, suggesting key biases in sampling effort within these taxa that have been the subject of much coronavirus research (Supplementary Table 9). Finally, members of several genera within the Pteropodinae subfamily were undersampled (that is, *Pteropus*, *Eidolon* and *Acerodon*), while others displayed greater sampling effort (that is, the subfamily Rousettinae).

### Heterogeneity in coronavirus infection prevalence

Using a phylogenetic meta-analysis model that accounted for sampling variance, bat phylogeny, additional species effects, and within- and between-study variation^127,128^, we observed high heterogeneity among coronavirus infection prevalence estimates (*I*^2^ = 84.2%, *Q*_1,854_ = 8,620.69, *P* < 0.0001). This heterogeneity was mainly due to within-study (43.65%) and between-study effects (31.53%), with smaller contributions from bat phylogeny (9.02%) and additional species effects (0.001%). When repeating this intercept-only model for alphacoronavirus- and betacoronavirus-specific datasets, prevalence showed similar patterns of heterogeneity (alphacoronavirus: *I*^2^ = 79.10%, *Q*_1,553_ = 4,973.72, *P* < 0.0001; betacoronavirus: *I*^2^ = 74.10%, *Q*_1,428_ = 3,871.49, *P* < 0.0001), mainly due to within-study (alphacoronavirus: 35.50%; betacoronavirus: 30.21%) and between-study effects (alphacoronavirus: 36.94%; betacoronavirus: 29.88%) and secondarily by phylogeny (alphacoronavirus: 6.66%; betacoronavirus: 14.02%) or other species-level effects (alphacoronavirus: 0.001%; betacoronavirus: 0%).

### Methodological and biological predictors of prevalence

**Fig. 4:**
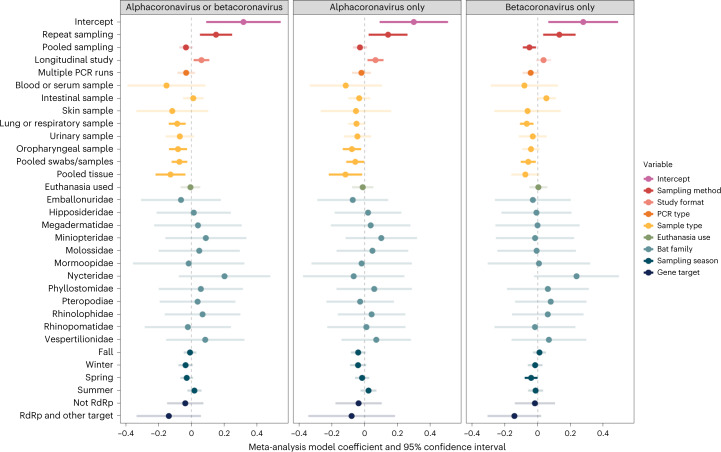
Methodological and biological predictors of coronavirus prevalence in wild bats. Phylogenetic meta-analysis model coefficients and 95% confidence intervals, estimated using REML for each of our three datasets. Colours indicate the nine variables included in each model (binary covariates for sampling season). Estimate confidence intervals are shaded by whether they cross zero (the vertical dashed line), with increased transparency denoting non-significant effects. The intercept contains the following reference levels: single sampling (sampling method); cross-sectional study (study format); single PCR (PCR type); faecal, rectal or anal sample (sample type); euthanasia not used (euthanasia use); Craseonycteridae (bat family); not fall, not winter, not spring and not summer (sampling season); and RNA-dependent RNA polymerase (RdRp) only (gene target). Sample sizes are 1,854 prevalence estimates for all coronaviruses, 1,553 prevalence estimates for only alphacoronaviruses and 1,428 prevalence estimates for only betacoronaviruses.

When considering the suite of methodological and biological predictors in our phylogenetic meta-analysis models, fixed effects explained approximately 20% of the variance in infection prevalence (pooled-coronavirus genera *R*^2^ = 0.19; alphacoronavirus-only *R*^2^ = 0.21; betacoronavirus-only *R*^2^ = 0.19). Sample type, sampling method and study format were the strongest predictors of coronavirus prevalence (Table 1). Within our pooled-coronavirus dataset, lung or respiratory samples (untransformed *β* = −0.09; 95% confidence interval (CI): −0.14 to −0.04, *P* = 0.001), oropharyngeal samples (untransformed *β* = −0.08; 95% CI: −0.14 to −0.03, *P* = 0.004), pooled swabs/samples (untransformed *β* = −0.07; 95% CI: −0.12 to −0.03, *P* = 0.003) and pooled tissue (untransformed *β* = −0.13; 95% CI: −0.22 to −0.04, *P* = 0.006) all had lower prevalence than faecal/rectal or intestinal samples, with weaker associations observed for only alphacoronaviruses and only betacoronaviruses (Fig. 4). Across all three datasets, repeat sampling was associated with a 0.70–1.6% increase in coronavirus prevalence (pooled coronavirus: untransformed *β* = 0.15; 95% CI: 0.05–0.25, *P* = 0.003; alphacoronavirus: untransformed *β* = 0.14; 95% CI: 0.03–0.26, *P* = 0.03; betacoronavirus: untransformed *β* = 0.13; 95% CI: 0.03–0.23, *P* = 0.009) as compared to one-time (single) sampling (Fig. 4). Similarly, longitudinal study design predicted a small increase (~0.23–0.33%) in positive viral detection in the pooled coronavirus (untransformed *β* = 0.06; 95% CI: 0.01–0.11, *P* = 0.01) and alphacoronavirus-only (untransformed *β* = 0.07; 95% CI: 0.02–0.12, *P* = 0.008) datasets, as opposed to cross-sectional sampling. Other model variables including sampling season, bat family, PCR type and gene target showed weak or no association with coronavirus positivity across all datasets. Notably, use of euthanasia was not associated with greater ability to detect coronavirus RNA (pooled coronavirus: untransformed *β* = −0.01; 95% CI: −0.07 to 0.05, *P* = 0.86; alphacoronavirus: untransformed *β* = −0.01; 95% CI: −0.08 to 0.05, *P* = 0.73; betacoronavirus: untransformed *β* = 0.004; 95% CI: −0.05 to 0.06, *P* = 0.89).

**Table 1:** Meta-analysis of coronavirus prevalence across studies

	Alphacoronavirus or betacoronavirus			Alphacoronavirus only			Betacoronavirus only		
	*Q*	*d.f.*	*P*	*Q*	*d.f.*	*P*	*Q*	*d.f.*	*P*
Sampling method	16.066	2	0.0003	9.347	2	0.0093	17.818	2	0.0001
Study format	6.302	1	0.0121	7.058	1	0.0079	2.252	1	0.1334
PCR type	1.368	1	0.2422	0.4157	1	0.5191	2.993	1	0.0837
Sample type	38.005	8	<0.0001	17.612	8	0.0243	30.033	8	0.0002
Euthanasia use	0.0332	1	0.8555	0.1166	1	0.7328	0.0186	1	0.8915
Bat family	11.5996	12	0.4783	10.8095	12	0.5453	14.9070	12	0.2466
Sampling season	8.3251	4	0.0804	9.9849	4	0.0407	6.9559	4	0.1382
Gene target	2.2751	2	0.3206	0.5962	2	0.7422	2.9593	2	0.2277

Analysis of variance (ANOVA) table from the phylogenetic meta-analysis model fit using REML to all data and each data subset (alphacoronavirus only or betacoronavirus only). For each variable, we provide Cochran’s *Q*, the associated degrees of freedom and the two-sided *P* value.

## Discussion

Since the onset of the COVID-19 pandemic, increased attention has been paid to bats as potential reservoir hosts of coronaviruses, presumably including viruses with zoonotic potential^129–131^. While other studies have reported data on the geographical and taxonomic distribution of reported bat hosts^131,132^, we generated a standardized, Preferred Reporting Items for Systematic Reviews and Meta-Analyses (PRISMA)-compliant open and static database of coronavirus surveillance in bats, which provides disaggregated data (including negative results). In doing so, our study takes an important step towards building an open database of wildlife disease surveillance with relevance to pandemic prediction and preparedness^133^.

Our database is a snapshot of bat coronavirus research before the COVID-19 pandemic and includes 110 studies, 2,274 records of infection prevalence and a total of 89,752 bat samples. Our geographic and taxonomic analyses reveal that most bat sampling has taken place in China, with gaps in surveillance in South Asia, the Americas, sub-Saharan and East Africa. Additionally, very few such studies were carried out in the United States and Canada.

Progress towards addressing gaps in surveillance has been made since the onset of the pandemic; for example, recent bat surveillance in Latin America and Madagascar has been reported^131,134–138^. Although phylogenetic coverage of bat species is a strength of the dataset, we identified taxonomic patterns in the intensity of sampling efforts. Our analyses confirm previous findings, such as a greater number of surveillance studies in the Rhinolophidae and a disproportionate number of studies in China^139^. However, we also characterized finer-scale variation in sampling effort relevant to prioritizing future surveillance. For example, although many studies have been conducted on rhinolophid bats, the Rhinolophidae and Hipposideridae families also had low sample sizes for coronavirus diagnostics, suggesting low power to detect viruses on a per-species basis. Further, subclades of the Hipposideridae and Rhinolophidae as well as the Rousettinae subfamily of pteropid bats were some of the most heavily sampled taxa versus considerable undersampling within subfamilies of phyllostomid bats in particular. Strengthening surveillance efforts in undersampled regions and specific bat taxa is important; for example, greater sampling of rhinolophid and hipposiderid species that fall outside identified well-sampled subclades is likely to uncover novel coronaviruses (Supplementary Table 9). Sampling the understudied Neotropical subfamilies Stenodermatinae and Glossophaginae might also have potential to uncover novel betacoronaviruses, as predicted by recent models^131^.

After controlling for bat phylogeny, sampling variance, and both study- and observation-level heterogeneity, we found that sample type, repeat sampling and longitudinal study design were the most important predictors of coronavirus prevalence. We did not find consistent support for seasonality in coronavirus prevalence^1,10^, whereas we did find support for longitudinal sampling enabling coronavirus detection^6,9^ and for successful coronavirus detection varying by sample type^11^. Specifically, lung or respiratory samples, urinary samples, oropharyngeal samples, pooled swabs and pooled tissue were associated with lower prevalence across all studies, with weaker effects generally observed in alphacoronavirus- and betacoronavirus-only datasets. In contrast, repeat sampling and longitudinal study designs, as well as intestinal and faecal and rectal samples, were consistently associated with viral detection. This might reflect gastrointestinal tropism of coronaviruses in bats^11^.

To optimize coronavirus detection, combining the above set of sampling approaches^140^, particularly using faecal samples or rectal swabs, should enhance detection of coronaviruses from wild bats. Moreover, longitudinal study designs will be crucial to pinpoint how coronaviruses are transmitted among wild bat hosts^140,141^ and identify the intrinsic and extrinsic drivers of virus shedding^142,142^. Euthanasia did not affect the likelihood of virus detection, which means that coronavirus surveillance can be accomplished with minimally invasive (for example, rectal swab) and readily accessible samples (for example, museum-derived, such as whole specimens or individual organs) rather than requiring terminal sampling^143^. Avoiding euthanasia reduces negative impacts of virus surveillance studies on bat population dynamics and enables longitudinal, mark-recapture designs. However, we note that selective terminal sampling can still provide other important benefits for virus surveillance, including the ability to post hoc confirm the species identity of voucher specimens, study tissue tropism and receptor usage of coronaviruses and provide lasting evidence of specific bat–virus associations in scientific collections^143,144^.

Our systematic review identified multiple challenges in synthesizing viral surveillance data from wildlife studies. Although study-level effects can be accounted for in part with random effects in meta-analysis, we note that at least some of our non-significant results could be due to variability in study format, sampling design and reporting. To reduce this limitation in the future, we encourage researchers to report data at the finest resolution possible (for example, fully stratified by location, timepoint, bat species, virus species or strain, and sample type). Developing and adopting data standards for reporting these types of data—and real-time channels to aggregate them with standardized metadata—could substantially improve our ability to address research questions regarding transmission dynamics, bat immunology, viral evolution and spillover risk.

## Methods

### Systematic review

To identify studies quantifying the proportion of wild bats positive for alpha- or betacoronaviruses using PCR or serological methods, we followed the PRISMA protocol (Fig. 1)^145^. We systematically searched Web of Science, PubMed and Global Health (a database comprising publications from the Public Health and Tropical Medicine database and CAB Abstracts). PubMed searches used the following string: (bat* OR Chiroptera*) AND (coronavirus* OR CoV*). Web of Science and Global Health (comprising CAB Abstracts and Public Health and Tropical Medicine database) searches used the following string: (bat* OR Chiroptera*) AND (coronavirus* OR CoV*) AND (wild*). Searches were performed on 24 September 2020 and included studies published in or after 1984.

We screened a total of 1,016 abstracts for studies that included sampling of wild bats for coronaviruses. Publications were excluded if they did not assess coronavirus prevalence in bats or were published in languages other than English (this led to the exclusion of only a single dissertation, written in Portuguese). In total, we identified a total of 159 candidate articles that we screened for these data. Of these, 110 studies tested bats for coronaviruses, reported reusable data and were included in our final, publicly available dataset. Geographic and taxonomic analyses, which did not rely on population-level prevalence estimates, were performed on a 108-study subset of the public dataset which excludes records with genus- or family-level versus species-level bat data and includes data that could not be used to calculate prevalence (for example, number of samples corresponds to geographic region rather than bat species). Infection prevalence analyses were performed on a 107-study subset of the public dataset. Each of these two datasets were then divided into three more: pooled-coronavirus genera (alphacoronaviruses and betacoronaviruses), alphacoronavirus genus-only and betacoronavirus genus-only (Supplementary Table 1). The datasets used for geographic and taxonomic analyses, which included data that could not be used to calculate prevalence (for example, number of samples corresponds to geographic region rather than bat species) had 37 (pooled-coronavirus genera), 21 (alphacoronavirus genus-only) and 9 (betacoronavirus genus-only) more rows than the corresponding infection prevalence datasets.

Our aim was to provide a comprehensive record of bat coronavirus surveillance up to the beginning of the COVID-19 pandemic, and our sample necessarily omits more recent publications that have reanalysed samples, motivated by investigations into the evolutionary origins of SARS-CoV-2 and other L2 lineage sarbecoviruses. It also omits the final dataset compiled by the USAID PREDICT dataset and released at the end of 2020. Standardized PREDICT format is a substantively different kind of data compared with all other studies we analysed; these data have been extensively analysed elsewhere^1^. Additionally, only 16 of the 110 studies in our database reported financial support from the PREDICT programme, suggesting that a substantial breadth of data collection exists in the literature beyond any one collaborative project.

### Data collection

Our initial dataset consists of a total of 110 studies and 2,274 records. Each record provides an infection prevalence estimate at the finest spatiotemporal, methodological and phylogenetic scale reported. More precisely, each unique record includes a distinct combination of coronavirus genus; bat genus, family and/or species; sample type; detection method (that is, PCR or serology); gene/protein target; date/sampling season and geographic location (sampling country, state, and specific site and/or geographic coordinates, if available). Sampling season was determined by month of sampling according to National Oceanic and Atmospheric Administration meteorological definitions; in the Northern Hemisphere, sample seasons equated to fall (September–November), winter (December–February), spring (March–May) and summer (June–August), while in the Southern Hemisphere these groupings were inverted (for example, December–February was classified as summer)^146^. Detection estimates derived at finer phylogenetic scales (for example, virus strain) were aggregated to genus. Prevalence estimates that combined two or more sample subtypes (for example, lung and small intestine) and that could not be further separated were recorded as pooled. As observed previously for bat filoviruses and henipaviruses, some studies pooled coronavirus detection estimates for more than one bat species^6^. Rows with these pooled prevalence estimates were excluded from subsequent statistical analyses. Study formats were classified as longitudinal and cross-sectional: prevalence estimates derived from repeated sampling at one location were marked as longitudinal, while those derived from one location on a specific date were listed as cross-sectional. Thus, most studies (92.7%) yielded more than one detection estimate record: for example, a longitudinal study that provides individual coronavirus detection estimates from two types of samples in a given bat species on six separate dates spanning several years would result in at least 12 records in the dataset.

In addition to these spatial and temporal components, we recorded data on detection methodology (for example, single or nested/multiple PCR for RNA detection or lateral flow immunoasssay for antigen detection), additional virus taxonomy (for example, subgenus, strain), PCR primers (and their gene targets) and whether the authors included information on the sex of the sampled bats or the use of euthanasia. We note that infection prevalence estimates are based on the number of samples tested for coronaviruses rather than the number of individual bats, as studies often tested multiple samples per individual specimen (for example, saliva, faeces, blood, tissue).

### Geographic and taxonomic analyses of sampling effort

With these data, we assessed geographic and taxonomic patterns in bat sampling effort. For the former, we fitted a GLM, with whether a country had been sampled for bat coronaviruses as a binomial response and region as the predictor in R. For sampled countries (*n* = 52), we fitted equivalent GLMs that modelled the number of unique studies and the total samples per country as a Poisson-distributed response. For each GLM, we assessed fit using McFadden’s *R*^2^ and the ‘performance’ package^147^. We also adjusted for the inflated false-discovery rate in post-hoc comparisons using ‘emmeans’^148^. Here and below, all statistical tests are two-tailed.

For taxonomic patterns, we derived equivalent response variables across bat species, using a recent phylogeny as a taxonomic backbone^15^. We note that despite being a recent synthesis, the number of bat species included this phylogeny (*n* = 1,287) remains an underestimate of known bat diversity (over 1,460 species); as such, corresponding taxonomic analyses necessarily exclude approximately 12% of extant bat species. Additionally, only four species in our dataset were absent from this phylogeny (*Pipistrellus taiwanesi*s, *Pipistrellus montanus*, *Myotis rufoniger*, *Rhinolophus cornutus*) and were excluded from phylogenetic analyses. We also reclassified species in the genus *Miniopterus* from the Vespertilionidae to be the sole members of the family Miniopteridae^149^. For all bat species in our phylogeny, we derived a binary response for whether a species had been sampled for coronaviruses. For those sampled species (*n* = 343), we derived the number of unique studies and the total samples. Using the ‘caper’ package^150^, we first estimated phylogenetic signal in sampling effort (that is, the propensity for related bat species to be sampled in a similar intensity). For binary sampling effort, we calculated *D*, where a value of 1 indicates a phylogenetically random trait distribution and 0 indicates phylogenetic clustering under a Brownian motion model of evolution^151^. For sampled species, we estimated Pagel’s *λ* for the log_10_-transformed number of studies and samples^152^. Next, we applied a graph-partitioning algorithm, phylogenetic factorization, to more flexibly identify any bat clades across taxonomic levels that differ in sampling effort. With a standardized taxonomy from our bat phylogeny^15^, we used the ‘phylofactor’ package to partition binary sampling effort, number of studies and number of samples in a series of iterative GLMs for each edge in the tree^16,153^. As in our geographic analyses, we modelled these variables with binomial and Poisson distributions. We then determined the number of significant clades using Holm’s sequentially rejective test with a 5% family-wise error rate^154^.

### Phylogenetic meta-analysis of infection prevalence

We first used the ‘metafor’ package to calculate Freeman–Tukey double arcsine-transformed proportions of coronavirus infection-positive bats and their corresponding sampling variances^10,18,20^. We then built two hierarchical meta-analysis models for three infection prevalence datasets: the global dataset, an alphacoronavirus-specific dataset and a betacoronavirus-specific dataset (see Supplementary Table 1 for the sample size per model). Each model was fitted using restricted maximum likelihood (REML) and included bat species and phylogeny (using the previous bat tree) as random effects alongside an observation-level random effect nested within a study-level effect^17^. The first model (that is, model 1) for each dataset only included an intercept and was used to estimate *I*^2^, which quantifies the contribution of true heterogeneity (rather than noise) to variance in infection prevalence^155^. We report both the overall *I*^2^ per dataset as well as the proportional *I*^2^ for each random effect, and we used Cochran’s *Q* to test whether such heterogeneity was greater than that expected by sampling error alone. The second model (that is, model 2) for each dataset included the following moderators: sampling method (repeat vs single), study format (longitudinal vs cross-sectional sampling), PCR type (nested/multiple vs single), sample analysed, whether terminal sampling was performed, bat family, sampling season and gene target. We calculated variance inflation factors for all moderators in the linear model; the moderators displayed no substantial collinearity^156^. To facilitate estimating model coefficients, we removed levels for any moderators with *n* < 3. For each iteration of model 2, we assessed moderator significance using the *Q* test (that is, a Wald-like test of all coefficients per moderator) and estimated a pseudo-*R*^2^ as the proportional reduction in the summed variance components compared against those from an intercept-only model^157^.

### Reporting summary

Further information on research design is available in the Nature Portfolio Reporting Summary linked to this article.

## Supplementary information

Supplementary Information Supplementary Tables 1–9.

Reporting Summary

## Data Availability

The primary dataset is available on GitHub (www.github.com/viralemergence/datacov; 10.5281/zenodo.6644163) and comprises data extracted from papers obtained during a systematic search of PubMed (https://pubmed.ncbi.nlm.nih.gov), Web of Science (https://www.webofscience.com) and Global Health (https://www.cabdirect.org/globalhealth). Source data are provided with this paper.

## Source data

Source Data Supplementary Table 1 Statistical source data.
Source Data Supplementary Table 2 Statistical source data.
Source Data Supplementary Tables 3, 4, 5, 8 and 9 Statistical source data.
Source Data Supplementary Table 6 Statistical source data.
Source Data Supplementary Table 7 Statistical source data.

## Code availability

Data were analysed in R Studio (v2021.9.2 ‘Ghost Orchid’). The unprocessed data and scripts to generate the primary dataset (and all other derived datasets) and to replicate all analyses and visualizations are available at www.github.com/viralemergence/batgap; 10.5281/zenodo.6644081.
