# Supplementary material for: Coronavirus sampling and surveillance in bats from 1996–2019: a systematic review and meta-analysis
Source: Nat Microbiol. 2023 May 25;8(6):1176–86. doi: 10.1038/s41564-023-01375-1 (PMC10234814; doi:10.1038/s41564-023-01375-1)
Supplement: Supplementary file 1 — Supplementary Tables 1–9. [file 41564_2023_1375_MOESM1_ESM.pdf]

nature microbiology

Analysis https://doi.org/10.1038/s41564-023-01375-1

Coronavirus sampling and surveillance in bats from 1996–2019: a systematic review and meta-analysis

In the format provided by the authors and unedited

Supporting Information:

Coronavirus sampling and surveillance in bats from 1996-2019: a systematic review and meta-analysis

Supplementary Table 1. Data included in each base meta-analytic model (fit using REML, intercept only). Levels of factors which appeared fewer than three times in the datasets (i.e., liver [tissue type]; Noctilionidae and Mystacinidae [bat family]) were not included in the full meta-analysis model with reported coefficients (fit using REML, see Figure 4).

**Viral genus (as reported by study)**	**Rows of available data**		**Included in models:**		
	**# rows (non-zero)**	**# rows (zero only)**	**CoV**	**α**	**β**
alphacoronavirus prevalence (explicit)	384	20	✓	✓	x
betacoronavirus prevalence (explicit)	240	26	✓	x	✓
genus unspecified or unresolved<br><br>(incl. if tests noted to include alphas and betas, but not specific, or if general sampling noted to include alpha and/or beta but not reported as proportion of sample)	39	1335*	✓	0	0
coinfection of any kind	6	0	x	x	x

*These rows comprise the zero prevalence estimates from studies using pan-coronavirus primers (most studies).

Supplementary Table 2. Distribution of sample types and associated percentages of positive and zero infection prevalence values.

	alphacoronavirus or betacoronavirus			alphacoronavirus only			betacoronavirus only		
sample type	*n*	% positive prevalence	% zero prevalence	n	% positive prevalence	% zero prevalence	*n*	% positive prevalence	% zero prevalence
fecal, rectal, or anal	1064	40.23	59.77	872	28.90	71.10	771	19.97	80.03
liver	2	100	0	0	-	-	2	100	0
blood or serum	3	0	100	3	0	100	3	0	100
intestine	184	39.67	60.33	144	22.92	77.08	145	23.45	76.55
lung or respiratory	74	9.46	90.54	66	6.06	93.94	70	4.29	95.71
oropharyngeal	106	8.49	91.51	100	7.00	93.00	98	2.04	97.96
skin swab	11	0	100	11	0	100	11	0	100
urinary	47	4.25	95.75	47	4.25	95.75	45	0	100
pooled swabs/samples	319	33.23	66.77	269	21.19	78.81	247	16.04	83.96
pooled tissue	49	28.57	71.43	44	20.45	79.55	40	12.50	87.50

Supplementary Table 3. Post-hoc analysis from the GLM (binomial errors) for binary sampling effort across countries globally as a function of geography. The *p*-values are two-tailed and are adjusted for multiple comparisons using the Benjamini-Hochberg correction.

	**Comparison**	**Odds ratio**	**SE**	**z ratio**	***p***
1	Africa / Americas	2.59	1.37	1.80	0.13
2	Africa / Asia	0.73	0.32	-0.73	0.59
3	Africa / Europe	0.73	0.32	-0.73	0.59
4	Africa / Oceania	4.11	3.26	1.78	0.13
5	Americas / Asia	0.28	0.15	-2.39	0.07
6	Americas / Europe	0.28	0.15	-2.39	0.07
7	Americas / Oceania	1.59	1.35	0.54	0.65
8	Asia / Europe	1.00	0.44	0	1
9	Asia / Oceania	5.63	4.47	2.17	0.07
10	Europe / Oceania	5.63	4.47	2.17	0.07

Supplementary Table 4. Post-hoc analysis from the GLM (Poisson errors) for the number of studies per country as a function of geography across all sampled countries. The *p*-values are two-tailed and are adjusted for multiple comparisons using the Benjamini-Hochberg correction.

	**Comparison**	**Risk ratio**	**SE**	**z ratio**	***p***
1	Africa / Americas	0.69	0.24	-1.04	0.61
2	Africa / Asia	0.40	0.10	-3.64	0.003
3	Africa / Europe	0.80	0.23	-0.76	0.64
4	Africa / Oceania	0.60	0.30	-1.03	0.61
5	Americas / Asia	0.57	0.18	-1.83	0.23
6	Americas / Europe	1.16	0.39	0.44	0.73
7	Americas / Oceania	0.87	0.46	-0.27	0.79
8	Asia / Europe	2.04	0.47	3.08	0.01
9	Asia / Oceania	1.52	0.71	0.90	0.62
10	Europe / Oceania	0.75	0.36	-0.60	0.68

Supplementary Table 5. Post-hoc analysis from the GLM (Poisson errors) for the number of samples tested per country as a function of geography across all sampled countries. The *p*-values are two-tailed and are adjusted for multiple comparisons via the Benjamini-Hochberg correction.

	**Comparison**	**Risk ratio**	**SE**	**z ratio**	***p***
1	Africa / Americas	0.91	0.01	-6.30	< 0.0001
2	Africa / Asia	0.41	0.004	-86.52	< 0.0001
3	Africa / Europe	1.91	0.03	44.72	< 0.0001
4	Africa / Oceania	0.83	0.02	-8.21	< 0.0001
5	Americas / Asia	0.45	0.01	-57.33	< 0.0001
6	Americas / Europe	2.11	0.04	43.40	< 0.0001
7	Americas / Oceania	0.91	0.02	-3.66	0.0003
8	Asia / Europe	4.64	0.06	119.77	< 0.0001
9	Asia / Oceania	2.01	0.04	32.30	< 0.0001
10	Europe / Oceania	0.43	0.01	-34.85	< 0.0001

Supplementary Table 6. Number of studies, number of samples analyzed, and percentage of dataset by global region.

	alphacoronavirus or betacoronavirus			alphacoronavirus only			betacoronavirus only		
global region	number of studies	number of samples analyzed	percentage of dataset (%)	number of studies	number of samples analyzed	percentage of dataset (%)	number of studies	number of samples analyzed	percentage of dataset (%)
Africa	17	15808	17.6	11	3863	15.1	14	2894	15.5
Americas	13	6896	7.7	12	4238	16.6	4	1034	5.5
Asia	56	56093	62.5	34	12242	47.9	41	13475	72.1
Australia or New Zealand	5	2277	2.5	4	1236	4.8	1	159	0.9
Europe	24	8678	9.7	17	3988	15.6	17	1115	6.0

Supplementary Table 7. Number of studies, number of samples analyzed, and percentage of dataset by global region and country.

	**alphacoronavirus or betacoronavirus**			**alphacoronavirus only**			**betacoronavirus only**		
global region: country	number of studies	number of samples analyzed	percentage of dataset (%)	number of studies	number of samples analyzed	percentage of dataset (%)	number of studies	number of samples analyzed	percentage of dataset (%)
Africa: Egypt	2	1507	1.68	0	0	0	2	1425	7.70
Africa: Gabon	1	1123	1.25	1	158	0.62	1	16	0.09
Africa: Ghana	3	7221	8.06	2	1904	7.45	2	225	1.22
Africa: Guinea	1	782	0.87	1	302	1.18	1	363	1.96
Africa: Kenya	3	2965	3.31	3	1192	4.66	3	490	2.65
Africa: Madagascar	2	1085	1.21	1	22	0.09	2	123	0.66
Africa: Mauritius	1	56	0.06	0	0	0	0	0	0
Africa: Morocco	1	15	0.02	1	3	0.01	0	0	0
Africa: Mozambique	1	264	0.29	1	226	0.88	1	14	0.08
Africa: Nigeria	1	79	0.09	0	0	0	1	79	0.43
Africa: Rwanda	1	541	0.6	1	22	0.09	1	158	0.85
Africa: Seychelles	1	50	0.06	0	0	0	0	0	0
Africa: South Africa	2	110	0.12	1	26	0.1	1	1	0.01
Africa: Tunisia	1	10	0.01	1	8	0.03	0	0	0
Americas: Brazil	4	957	1.07	3	547	2.14	2	90	0.49
Americas: Canada	3	442	0.49	3	442	1.73	0	0	0
Americas: Costa Rica	1	404	0.45	1	162	0.63	0	0	0

Americas: Mexico	2	3922	4.38	2	2571	10.06	2	944	5.10
Americas: Trinidad	1	112	0.13	1	14	0.05	0	0	0
Americas: USA	2	1059	1.18	2	502	1.96	0	0	0
Asia: Cambodia	1	173	0.19	1	64	0.25	1	63	0.34
Asia: China	32	45430	50.72	19	9999	39.11	28	11102	60.00
Asia: India	1	668	0.75	0	0	0	1	341	1.84
Asia: Indonesia	2	80	0.09	0	0	0	1	17	0.09
Asia: Japan	2	158	0.18	1	120	0.47	1	27	0.15
Asia: Laos	1	59	0.07	0	0	0	0	0	0
Asia: Lebanon	1	451	0.5	0	0	0	1	441	2.39
Asia: Malaysia	1	365	0.41	0	0	0	0	0	0
Asia: Myanmar	1	666	0.74	1	108	0.42	0	0	0
Asia: Philippines	2	283	0.32	2	5	0.02	2	209	1.13
Asia: Saudi Arabia	1	1262	1.41	1	532	2.08	1	259	1.40
Asia: Singapore	1	1555	1.74	0	0	0	0	0	0
Asia: South Korea	2	684	0.76	1	4	0.02	2	83	0.45
Asia: Sri Lanka	1	50	0.06	0	0	0	1	50	0.27
Asia: Taiwan	4	625	0.7	3	219	0.86	1	50	0.27
Asia: Thailand	3	2812	3.14	3	898	3.51	3	615	3.32
Asia: Vietnam	2	593	0.66	2	293	1.15	1	45	0.24
Australia	4	2273	2.54	3	1232	4.82	1	159	0.86
New Zealand	1	4	0	1	4	0.02	0	0	0

Europe: Belgium	1	142	0.16	0	0	0	0	0	0
Europe: Bulgaria	1	1201	1.34	1	743	2.91	1	383	2.07
Europe: Denmark	1	271	0.3	1	213	0.83	0	0	0
Europe: Finland	1	78	0.09	1	38	0.15	1	22	0.11
Europe: France	4	1896	2.12	4	504	1.97	0	0	0
Europe: Germany	4	1635	1.83	3	1209	4.73	0	0	0
Europe: Hungary	1	447	0.5	1	168	0.66	1	3	0.02
Europe: Italy	6	1197	1.34	2	463	1.81	6	352	1.90
Europe: Luxembourg	1	254	0.28	1	88	0.34	1	77	0.42
Europe: Netherlands	2	225	0.25	1	177	0.69	1	8	0.04
Europe: Romania	1	172	0.19	0	0	0	1	172	0.93
Europe: Slovenia	1	106	0.12	0	0	0	1	36	0.19
Europe: Spain	2	923	1.03	2	323	1.26	2	56	0.30
Europe: Ukraine	1	19	0.02	0	0	0	1	6	0.03
Europe: UK	1	112	0.12	1	62	0.24	0	0	0

Supplementary Table 8. Results of phylogenetic factorization applied to the number of studies per sampled bat species (Poisson GLM). The number of retained phylogenetic factors (following a 5% family-wise error rate applied to GLMs), taxa corresponding to those clades, number of species, and mean number of studies for the clade compared to the paraphyletic remainder are shown. Bat taxonomy follows Upham et al. 2019.

factor	taxa	tips	node	clade	other
1	*Myotis escalerai, Myotis pequinius, Myotis chinensis, Myotis nattereri, Myotis punicus, Myotis myotis, Myotis blythii, Myotis bechsteinii, Myotis petax, Myotis macrodactylus, Myotis fimbriatus, Myotis pilosus, Myotis daubentonii*	13	495	6.93	2.84
2	*Pipistrellus nanulus, Pipistrellus nathusii, Pipistrellus pygmaeus, Pipistrellus pipistrellus, Pipistrellus hesperidus, Pipistrellus kuhlii, Pipistrellus deserti, Pipistrellus tenuis, Pipistrellus javanicus, Pipistrellus abramus, Nyctalus*	15	392	6.00	2.86
3	Rhinolophidae	35	593	4.49	2.82

Supplementary Table 9. Results of phylogenetic factorization applied to the number of tested samples per sampled bat species (Poisson GLM). The number of retained phylogenetic factors (following a 5% family-wise error rate applied to GLMs), taxa corresponding to those clades, number of species, and mean number of tested samples for the clade compared to the paraphyletic remainder are shown. Bat taxonomy follows Upham et al. 2019.

factor	taxa	tips	node	clade	other
1	*Rhinolophus osgoodi, Rhinolophus affinis, Rhinolophus coelophyllus, Rhinolophus shameli, Rhinolophus stheno, Rhinolophus rouxii, Rhinolophus thomasi, Rhinolophus sinicus, Rhinolophus megaphyllus, Rhinolophus borneensis, Rhinolophus lepidus, Rhinolophus pusillus, Rhinolophus rex, Rhinolophus macrotis, Rhinolophus malayanus, Rhinolophus rufus*	16	609	1522.56	183.45
2	*Hipposideros cervinus, Hipposideros pomona, Hipposideros cineraceus, Hipposideros ater, Hipposideros halophyllus, Hipposideros dyacorum, Hipposideros ruber, Hipposideros abae, Hipposideros caffer, Hipposideros fuliginosus*	10	635	971.60	224.13
3	*Eonycteris, Rousettus, Epomops, Epomophorus, Micropteropus, Nanonycteris, Hypsignathus, Myonycteris*	14	667	541.86	233.33
4	Miniopteridae	16	519	486.81	234.13
5	*Myotis escalerai, Myotis pequinius, Myotis chinensis, Myotis nattereri, Myotis punicus, Myotis myotis, Myotis blythii, Myotis bechsteinii, Myotis petax, Myotis macrodactylus, Myotis fimbriatus, Myotis pilosus, Myotis daubentonii*	13	495	401.69	239.78
6	Mystacinidae, Noctilionidae, Mormoopidae, Phyllostomidae, Molossidae, Vespertilionidae, Nycteridae, Emballonuridae	210	345	85.94	498.52
7	*Pipistrellus nanulus, Pipistrellus nathusii, Pipistrellus pygmaeus, Pipistrellus pipistrellus, Pipistrellus hesperidus,*	15	392	235.73	246.38

	Pipistrellus kuhlii, Pipistrellus deserti, Pipistrellus tenuis, Pipistrellus javanicus, Pipistrellus abramus, Nyctalus				
8	*Centurio, Artibeus, Dermanura, Vampyressa, Platyrrhinus, Uroderma, Sturnira, Carollia*	21	552	175.10	250.54
9	*Rhinolophus capensis, Rhinolophus simulator, Rhinolophus denti, Rhinolophus blasii, Rhinolophus euryale, Rhinolophus mehelyi, Rhinolophus mossambicus, Rhinolophus hildebrandtii, Rhinolophus darlingi, Rhinolophus ferrumequinum, Rhinolophus clivosus*	11	596	324.00	243.33
10	*Glossophaga, Leptonycteris, Anoura, Hylonycteris, Choeroniscus, Trachops, Mimon, Phyllostomus, Tonatia, Phylloderma, Lophostoma, Chrotopterus*	16	542	12.19	257.35
11	*Coelops, Aselliscus, Hipposideros*	12	631	256.50	246.53
12	*Eidolon, Pteropus, Acerodon*	11	657	225.64	246.59
13	*Neoromicia, Pipistrellus, Nyctophilus*	14	411	19.07	255.57
14	*Vespertilio, Tylonycteris, Nycticeinops, Vespadelus, Chalinolobus*	10	406	160.70	248.48
15	*Corynorhinus, Euderma, Plecotus, Barbastella, Rhogeessa, Bauerus, Antrozous, Lasiurus, Parastrellus*	18	440	25.89	258.10
16	*Scoteanax, Kerivoula, Harpiola, Murina, Scotorepens*	12	462	22.33	254.02
17	*Scotophilus, Glauconycteris, Eptesicus, Lasionycteris, Ia, Scotomanes*	18	387	114.17	253.22
18	Nycteridae, Emballonuridae	13	347	113.54	251.13
19	*Myotis aurascens, Myotis ikonnikovi, Myotis altarium, Myotis dasycneme, Myotis muricola, Myotis secundus, Myotis occultus, Myotis horsfieldii, Myotis macropus, Myotis adversus, Myotis capaccinii, Myotis longipes, Myotis laniger, Myotis siligorensis, Myotis davidii*	15	492	46.53	255.04

20	Molossidae, Vespertilionidae	45	359	70.69	272.38
21	*Mormopterus beccarii, Mormopterus norfolkensis, Tadarida brasiliensis, Otomops, Nyctinomops, Molossops, Cynomops, Molossus, Eumops, Tadarida teniotis, Tadarida australis, Mormopterus acetabulosus*	17	368	51.71	256.05
22	*Myotis mystacinus, Myotis alcathoe, Myotis elegans, Myotis riparius, Myotis keaysi, Myotis yumanensis, Myotis velifer, Myotis nigricans, Myotis brandtii, Myotis volans, Myotis ciliolabrum, Myotis californicus, Myotis lucifugus, Myotis evotis, Myotis thysanodes*	15	475	59.40	255.11
23	Rhinolophidae, Hipposideridae	12	592	75.00	252.11
24	*Rhinolophus rouxii, Rhinolophus thomasi, Rhinolophus sinicus, Rhinolophus megaphyllus, Rhinolophus borneensis, Rhinolophus lepidus, Rhinolophus pusillus, Rhinolophus rex, Rhinolophus macrotis, Rhinolophus malayanus*	10	610	2005.80	193.07
25	*Rhinolophus rouxii, Rhinolophus thomasi, Rhinolophus sinicus*	3	611	5509	199.48
26	*Rhinolophus rouxii*	1	22	12	246.60
27	*Rhinolophus sinicus*	1	24	16499	198.39
28	*Hipposideros pomona, Hipposideros cineraceus, Hipposideros ater, Hipposideros halophyllus, Hipposideros dyacorum, Hipposideros ruber, Hipposideros abae, Hipposideros caffer, Hipposideros fuliginosus*	9	636	1071.11	223.68
29	*Hipposideros pomona, Hipposideros cineraceus, Hipposideros ater, Hipposideros halophyllus, Hipposideros dyacorum*	5	637	571.20	241.11
30	*Hipposideros pomona, Hipposideros cineraceus, Hipposideros ater, Hipposideros halophyllus*	4	638	705.50	241.11
31	*Hipposideros pomona, Hipposideros*	2	639	1380	239.27

	cineraceus				
32	*Hipposideros cineraceus*	1	55	34	246.54
33	*Eonycteris*	1	77	1461	242.37
34	*Epomops, Epomophorus, Micropteropus, Nanonycteris, Hypsignathus, Myonycteris, Rousettus lanosus*	9	669	61.56	250.89
35	*Epomops, Epomophorus, Micropteropus, Nanonycteris, Hypsignathus, Myonycteris*	8	670	68.50	250.16
36	*Myonycteris*	1	89	139	246.23
37	*Epomops, Epomophorus, Micropteropus, Nanonycteris*	6	672	67.17	249.10
38	*Epomops*	1	82	3	246.63
39	*Epomophorus, Micropteropus*	4	674	99.75	247.64
