# Supplementary material for: Coronavirus sampling and surveillance in bats from 1996–2019: a systematic review and meta-analysis
Source: Nat Microbiol. 2023 May 25;8(6):1176–86. doi: 10.1038/s41564-023-01375-1 (PMC10234814; doi:10.1038/s41564-023-01375-1)
Supplement: Supplementary file 2 — Reporting Summary [file 41564_2023_1375_MOESM2_ESM.pdf]

nature portfolio

Corresponding author(s): Lily E. Cohen

Last updated by author(s): Mar 10, 2023

Reporting Summary

Nature Portfolio wishes to improve the reproducibility of the work that we publish. This form provides structure for consistency and transparency in reporting. For further information on Nature Portfolio policies, see our Editorial Policies and the Editorial Policy Checklist.

Statistics

For all statistical analyses, confirm that the following items are present in the figure legend, table legend, main text, or Methods section.

n/a	Confirmed	
☐	☒	The exact sample size (*n*) for each experimental group/condition, given as a discrete number and unit of measurement
☐	☒	A statement on whether measurements were taken from distinct samples or whether the same sample was measured repeatedly
☐	☒	The statistical test(s) used AND whether they are one- or two-sided<br>*Only common tests should be described solely by name; describe more complex techniques in the Methods section.*
☐	☒	A description of all covariates tested
☐	☒	A description of any assumptions or corrections, such as tests of normality and adjustment for multiple comparisons
☐	☒	A full description of the statistical parameters including central tendency (e.g. means) or other basic estimates (e.g. regression coefficient) AND variation (e.g. standard deviation) or associated estimates of uncertainty (e.g. confidence intervals)
☐	☒	For null hypothesis testing, the test statistic (e.g. *F*, *t*, *r*) with confidence intervals, effect sizes, degrees of freedom and *P* value noted<br>*Give P values as exact values whenever suitable.*
☒	☐	For Bayesian analysis, information on the choice of priors and Markov chain Monte Carlo settings
☐	☒	For hierarchical and complex designs, identification of the appropriate level for tests and full reporting of outcomes
☐	☒	Estimates of effect sizes (e.g. Cohen's *d*, Pearson's *r*), indicating how they were calculated

Our web collection on statistics for biologists contains articles on many of the points above.

Software and code

Policy information about availability of computer code

Data collection	Data was collected in Airtable (version 1.5.3) and RStudio (version 2021.9.2 "Ghost Orchid"). The unprocessed data and scripts to generate the primary dataset (and all other derived datasets) are available at www.github.com/viralemergence/batgap; DOI: 10.5281/zenodo.6644081.
Data analysis	Data was analyzed in RStudio (version 2021.9.2 "Ghost Orchid"). The unprocessed data and scripts to generate the primary dataset (and all other derived datasets) and to replicate all analyses and visualizations are available at www.github.com/viralemergence/batgap; DOI: 10.5281/zenodo.6644081).

For manuscripts utilizing custom algorithms or software that are central to the research but not yet described in published literature, software must be made available to editors and reviewers. We strongly encourage code deposition in a community repository (e.g. GitHub). See the Nature Portfolio guidelines for submitting code & software for further information.

Data

Policy information about availability of data

All manuscripts must include a data availability statement. This statement should provide the following information, where applicable:

- Accession codes, unique identifiers, or web links for publicly available datasets
- A description of any restrictions on data availability
- For clinical datasets or third party data, please ensure that the statement adheres to our policy

The primary dataset is available on Github (www.github.com/viralemergence/datacov; DOI: 10.5281/zenodo.6644163) and is comprised of data extracted from

papers obtained during a systematic search of PubMed (https://pubmed.ncbi.nlm.nih.gov), Web of Science (https://www.webofscience.com), and Global Health (https://www.cabdirect.org/globalhealth).

Human research participants

Policy information about studies involving human research participants and Sex and Gender in Research.

Reporting on sex and gender	N/A
Population characteristics	N/A
Recruitment	N/A
Ethics oversight	N/A

Note that full information on the approval of the study protocol must also be provided in the manuscript.

Field-specific reporting

Please select the one below that is the best fit for your research. If you are not sure, read the appropriate sections before making your selection.

☐ Life sciences ☐ Behavioural & social sciences ☒ Ecological, evolutionary & environmental sciences

For a reference copy of the document with all sections, see nature.com/documents/nr-reporting-summary-flat.pdf

Ecological, evolutionary & environmental sciences study design

All studies must disclose on these points even when the disclosure is negative.

Study description	Following the Preferred Reporting Items for Systematic Reviews and Meta-Analyses (PRISMA) protocol, we generate and analyze the first standardized, open database of coronavirus surveillance in bats that provides disaggregated prevalence data (including negative results) at the finest methodological, spatiotemporal, and phylogenetic level of detail possible from public records.
Research sample	A database with over 2,200 estimates of coronavirus infection prevalence or seroprevalence in bats from 110 published studies and 89,752 samples.
Sampling strategy	We followed the PRISMA protocol (Figure 1). We systematically searched Web of Science, PubMed, and Global Health (a database comprising publications from the Public Health and Tropical Medicine database and CAB Abstracts). PubMed searches used the following string: (bat* OR Chiroptera*) AND (coronavirus* OR CoV*). Web of Science and Global Health (comprised of CAB Abstracts and Public Health and Tropical Medicine database) searches used the following string: (bat* OR Chiroptera*) AND (coronavirus* OR CoV*) AND (wild*). Searches were performed on September 24, 2020.
Data collection	L.E.C., A.F., and B.C. collected the data from the primary literature and compiled it in a shared Airtable.
Timing and spatial scale	The database includes studies published between 2005 and 2020. The spatial scale is global.
Data exclusions	Initially, studies were excluded if they did not provide estimate(s) of bat coronavirus prevalence or seroprevalence (e.g., authors only report detection of a novel sequence but not the total bats sampled nor number positive); this criterion was pre-established. During data collection, we excluded studies that provided contradictory data that could not be resolved through communication with authors and studies which did not provide either bat genus or species information. Numbers of studies and records excluded are provided in the PRISMA (Figure S1).
Reproducibility	Unprocessed data and scripts to generate the primary dataset and to reproduce all analyses and visualizations are provided at www.github.com/viralemergence/batgap; DOI: 10.5281/zenodo.6644081.
Randomization	Study inclusion followed a systematic inclusion and exclusion process and can be considered a random sample of the literature.
Blinding	Blinding was not relevant to the meta-analysis or included in the PRISMA protocol.

Did the study involve field work? ☐ Yes ☒ No

Reporting for specific materials, systems and methods

We require information from authors about some types of materials, experimental systems and methods used in many studies. Here, indicate whether each material, system or method listed is relevant to your study. If you are not sure if a list item applies to your research, read the appropriate section before selecting a response.

Materials & experimental systems

n/a	Involved in the study
☒	☐ Antibodies
☒	☐ Eukaryotic cell lines
☒	☐ Palaeontology and archaeology
☒	☐ Animals and other organisms
☒	☐ Clinical data
☒	☐ Dual use research of concern

Methods

n/a	Involved in the study
☒	☐ ChIP-seq
☒	☐ Flow cytometry
☒	☐ MRI-based neuroimaging
